# Application of Functionalized DVB-*co*-GMA Polymeric Microspheres in the Enhanced Sorption Process of Hazardous Dyes from Dyeing Baths

**DOI:** 10.3390/molecules25225247

**Published:** 2020-11-11

**Authors:** Monika Wawrzkiewicz, Beata Podkościelna, Przemysław Podkościelny

**Affiliations:** 1 Department of Inorganic Chemistry, Institute of Chemical Sciences, Faculty of Chemistry, Maria Curie-Sklodowska University in Lublin, M. Curie-Sklodowska Sq. 3, 20-031 Lublin, Poland; 2 Department of Polymer Chemistry, Institute of Chemical Sciences, Faculty of Chemistry, Maria Curie-Sklodowska University in Lublin, M. Curie-Sklodowska Sq. 3, 20-031 Lublin, Poland; beatapod@poczta.umcs.lublin.pl; 3 Faculty of Chemistry, Maria Curie Sklodowska University in Lublin, M. Curie-Sklodowska Sq. 3, 20-031 Lublin, Poland; przemyslaw.podkoscielny@poczta.umcs.lublin.pl

**Keywords:** polymeric resin, dye removal, sorption, Acid Violet 1, Acid Green 16, Acid Red 18

## Abstract

Intensive development of many industries, including textile, paper, plastic or food, generate huge amounts of wastewaters containing not only toxic dyes but also harmful auxiliaries such as salts, acid, bases, surfactants, oxidants, heavy metal ions. The search for effective pollutant adsorbents is a huge challenge for scientists. Synthesis of divinylbenzene copolymer with glycidyl methacrylate functionalized with triethylenetetramine (DVB-*co*-GMA-TETA) resin was performed and the obtained microspheres were evaluated as a potential adsorbent for acid dye removal from dyeing effluents. The sorption capacities were equal to 142.4 mg/g for C.I. Acid Green 16 (AG16), 172 mg/g for C.I. Acid Violet 1 (AV1) and 216.3 mg/g for C.I. Acid Red 18 (AR18). Non-linear fitting of the Freundlich isotherm to experimental data was confirmed rather than the Langmuir, Temkin and Dubinin-Radushkevich. The kinetic studies revealed that intraparticle diffusion is the rate-limiting step during dye adsorption. Auxiliaries such as Na_2_SO_4_ (5–25 g/L), CH_3_COOH (0.25–1.5 g/L) and anionic surfactant (0.1–0.5 g/L) present in the dyeing baths enhance the dye adsorption by the resin in most cases. Regeneration of DVB-*co*-GMA-TETA is possible using 1 M NaCl-50% *v/v* CH_3_OH.

## 1. Introduction

Due to industrialization and significant consumerism in the world, often not meeting the assumptions consistent with the concept of green chemistry, it is reasonable to improve the wastewater purification technologies. It allows the assessment of the quality of the environment in order to protect human health. Various methods are used to remove organic and inorganic pollutants, such as precipitation, extraction, osmosis, filtration and oxidation, for example. Sorption is one of the most popular and widely used techniques. This process consists of concentrating the dissolved contaminant by transferring it to the sorbent phase. The efficiency of the process is closely related to the physicochemical properties of the sorbent (it depends, inter alia, on the type and amount of active centers/functional groups, structure, surface area and the energy of particle interaction). The universality of the method is related to its simplicity and it is used primarily in the development of effective procedures for removing various pollutants, which in the times of heavy industrialization constitute a serious problem for the environment. The use of the sorption process in environmental protection is usually carried out using the dynamic method, i.e., columns packed with an adsorbent. Industrial application is preceded by laboratory tests in the batch system. In addition to the already known adsorbents, more effective and appropriate materials are being sought. The popular sorbents such as activated carbons [1], zeolites [2,3], low-cost adsorbents [4,5,6], ion exchange resins [7,8], have found application in dye removal technologies. Dyes are widely used in the textile, chemical and cellulose industries, for the production of plastics, paints and varnishes. Over 10,000 different dyes and more than 700,000 tons of dyes are produced every year, and 2–50% are discharged into the aquatic environment [9,10]. Many dyes, especially those containing azo groups or aromatic rings, e.g., acid type dyes, discharged into water as a waste can causes serious danger as they are difficult to degrade [9]. Moreover, some of them are mutagenic and carcinogenic [9].

This study presents synthesis and application of the functionalized polymeric resin DVB-*co*-GMA-TETA for the removal of three textile dyes (C.I. Acid Violet 1, Acid Green 16, C.I. Acid Red 18) from aqueous solutions. Physicochemical characteristics of DVB-*co*-GMA-TETA were determined using different techniques, e.g., Fourier transform infrared spectroscopy (FT-IR) with attenuated total reflectance mode (ATR), nitrogen adsorption/desorption analysis, elemental analysis and optical microscopy. The impacts of parameters such as dyes–microspheres phase contact time, dye initial concentration and auxiliaries (Na_2_SO_4_, CH_3_COOH, anionic surfactant sodium dodecyl sulfate (SDS)) concentration were evaluated on dye sorption effectiveness on the modified polymeric resin. Desorption of dyes from the resin was also studied.

## 2. Results

### 2.1. DVB-co-GMA-TETA Synthesis and Preliminary Characteristics

**Figure 1:**
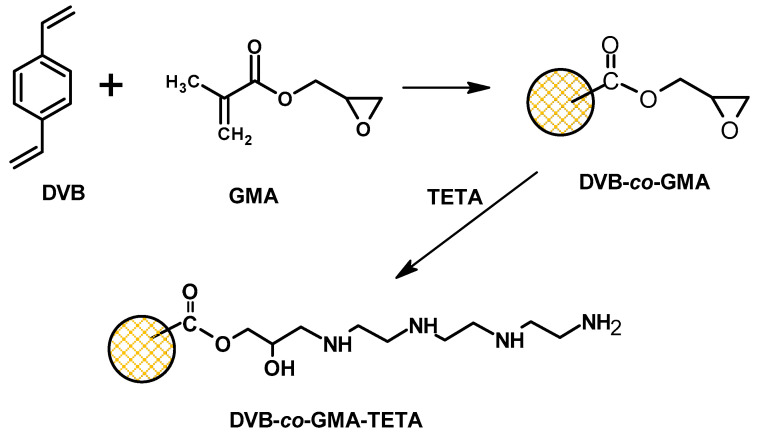
Chemical structure and functionalization of microspheres.

Figure 1 presents the chemical structure of monomers and synthesis of the amine derivative. For the synthesis of porous polymers in spherical form, the suspension polymerization method was applied [11,12,13,14]. The use of glycidyl methacrylate, a monomer that possesses two functional groups (methacrylic and epoxy), leads to the obtention of polymeric microspheres with reactive sites on the surface, capable for further modification. Epoxides in the presence of amine are subject to a ring-opening reaction with good performance, which has been confirmed by elemental analysis. The elemental composition of DVB-*co*-GMA-TETA is the following: 67.08% C, 7.68% H and 5.8% N.

In Table 1, characteristic parameters of the porous structures of the parent DVB-*co*-GMA microspheres are presented. The specific surface area (S_BET_) and total pore volume (V_TOT_) for the DVB-*co*-GMA copolymer are 152 m^2^/g and 0.524 cm^3^/g, respectively. The average pore diameter has a value of 13.82 nm whereas the most probable pore diameter possesses two maxima, 4 and 20 nm. The obtained microspheres can be classified as mesoporous materials. We observed a decrease of S_BET_ and an increase of V_TOT_ after functionalization. The obtained data were compared with those available in the literature for the commercially available polystyrene-divinylbenzene (PS-DVB) resin Amberlite XAD-4 and polyacrylic-divinylbenzene (PA-DVB) resin Amberlite XAD-7, nano-conducting polymer composites and bentonite (Table 1).

**Figure 2:**
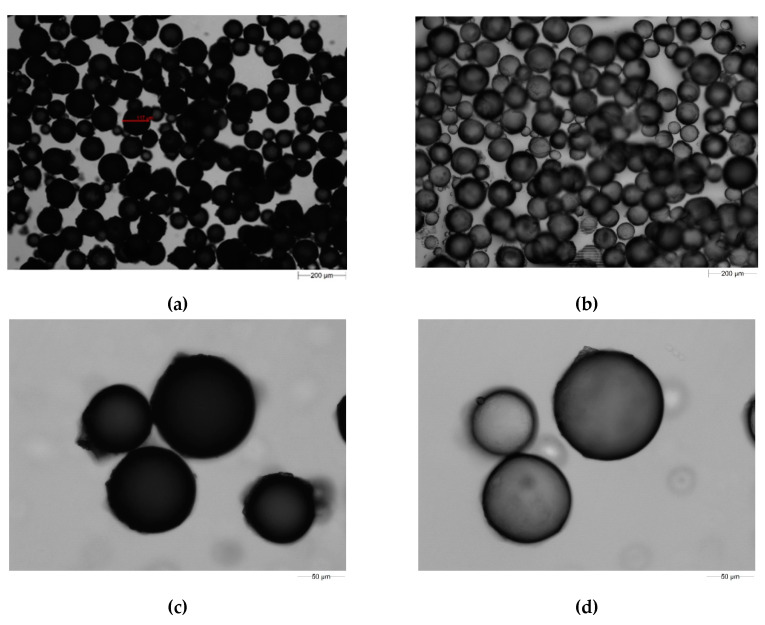
Photos of the synthesized microspheres (**a**,**c**) and (**b**,**d**) those swollen with acetone obtained using optical microscope Morphologi G3.

In Figure 2a,c, photos of the microspheres obtained using optical microscope are presented. As one can see, copolymers possess a spherical shape which influences, among others, to reduce the flow resistance. Photos of the microspheres, after swelling with acetone, were also taken (Figure 2b,d), and the diameter of the microspheres increased by approximately 20%.

#### ATR-FT-IR Spectroscopy

**Figure 3:**
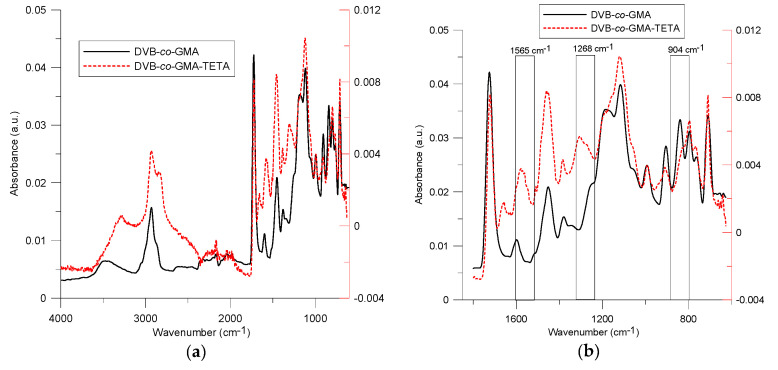
ATR-FT-IR spectra of DVB-*co*-GMA and DVB-*co*-GMA-TETA copolymers in the ranges: (**a**) 4000–750 cm^−1^ and (**b**) 600–1800 cm^−1^.

The ATR-FT-IR spectra of the parent DVB-*co*-GMA copolymers, and after modification with TETA, are presented in Figure 3a,b. In the both spectra, C-H stretching vibrations of -CH_2_- groups are observed at 2930–2932 cm^−1^. The vibrations of the -OH group are visible at 3474 cm^−1^. The intense bands at 1724 cm^−1^ were assigned to the stretching vibrations of carbonyl groups C=O ester moieties. The epoxide group in the DVB-*co*-GMA spectrum gives a shape signal at 905 cm^−1^. The differences in the signals before and after the modification are clearly visible and additionally marked in Figure 3b in the range 600–1800 cm^−1^. In the spectrum, the vibrations of N-H groups are visible at 1565 cm^−1^. The signal at 1268 cm^−1^ corresponds to C-N vibrations from amine molecules. The disappearance of signal from the epoxy group in the spectrum DVB-*co*-GMA-TETA indicates the correct course of the modification reaction.

**Figure 4:**
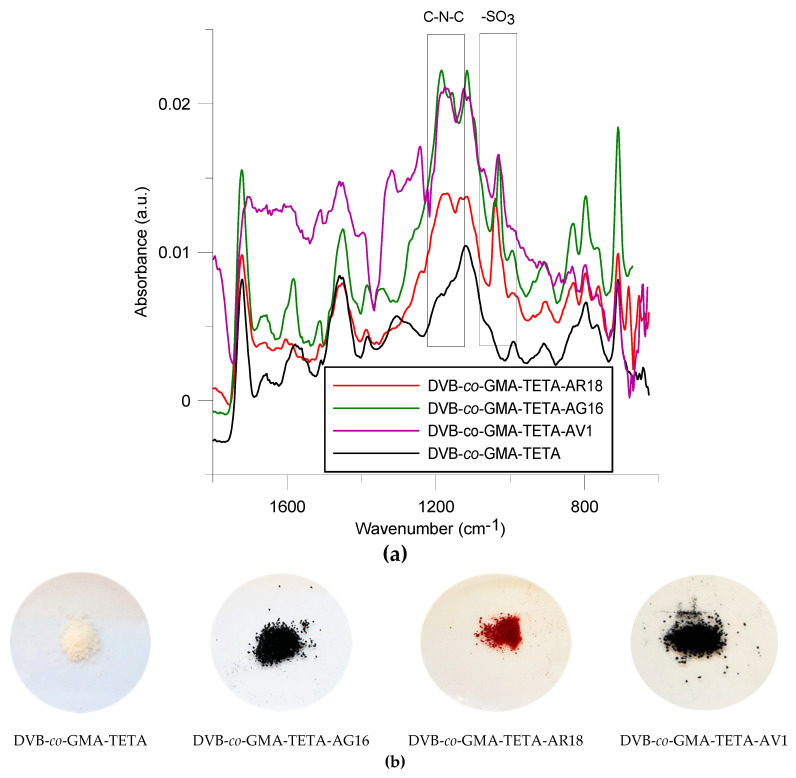
ATR-FT-IR spectra of DVB-*co*-GMA-TETA before and after dye sorption (**a**) as well as (**b**) photos of the microspheres before and after the sorption process.

In Figure 4, ATR-FT-IR spectra of the microsphere before and after the sorption process are presented. All spectra look very similar. The signals are weakened due to strong coloration of the microspheres. After the sorption process, the appearance of a new signal can be observed, compared to the original sorbent (DVB-*co*-GMA-TETA), in the range 1170–1190 cm^−1^, corresponding probably to C-N stretching vibrations from sec-aliphatic amines, and about 1040 cm^−1^ corresponding to symmetrical -SO_3_ stretching vibrations. 

### 2.2. Determination of DVB-co-GMA-TETA Sorption Capacity

The amounts of AV1, AG16 and AR18 sorbed by DVB-*co*-GMA-TETA microspheres at equilibrium, commonly known as the sorption capacities (*q_e_*), were calculated from Equation (1):

(1) $$q_{e}=\;\frac{\left(C_{0}-\;C_{e}\right)}{m}\;V$$

where *C*_0_ and *C_e_* (mg/L) are acid dye concentrations in the solution before adsorption and at equilibrium, respectively; *V* (L) is volume of the dye solution and *m* (g) is mass of DVB-*co*-GMA-TETA.

The Langmuir, Freundlich, Temkin and Dubinin-Radushkevich isotherm models (Table 2, Equations (2)–(5)) were selected to illustrate the relationship between the residual concentration of AV1, AG16 and AR18 dyes in aqueous phase at equilibrium and the sorption capacity of DVB-*co*-GMA-TETA at a constant room temperature. Characteristic parameters of selcected isotherm models as well as calculated Marquardt’s percent standard deviation (MPSD), determination coefficient (*R*^2^) and adjusted R-squared ($R_{adj}^{2}$), which are essential for the evaluation of the best fitting model in a given sorption system, are presented in Table 3 [20].

**Figure 5:**
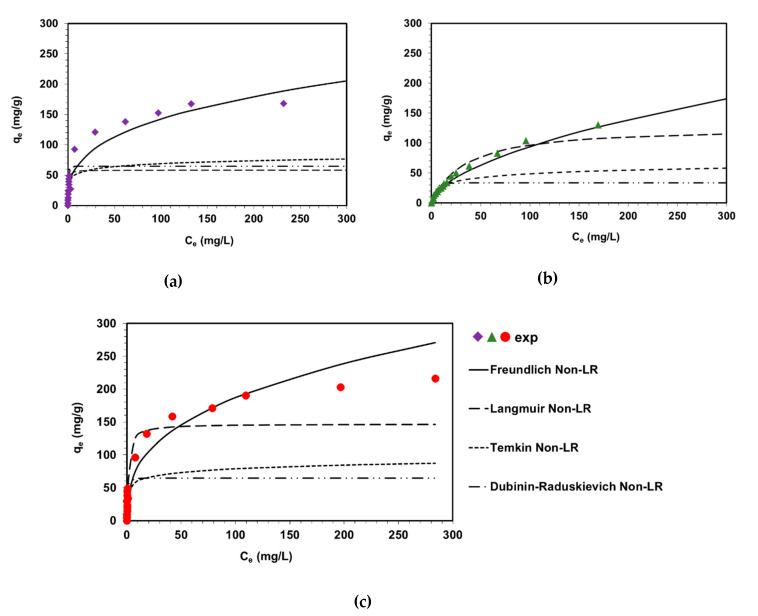
Adsorption isotherms of AV1 (**a**), AG16 (**b**) and AR18 (**c**) on DVB-*co*-GMA-TETA and fitting of the Langmuir, Freundlich, Temkin and Dubinin-Raduskievich to experimental data using non-linear regression (Non-LR).

The maximum values of sorption capacities determined experimentally (*Q_e exp_*) were found to be 142.4 mg/g for AG16, 172.0 mg/g for AV1 and 216.3 mg/g for AR18 (Figure 5). Based on obtained data shown in Table 3 and Figure 5, the highest *R*^2^ and $R_{adj}^{2}$ values—among four applied models—were obtained using the Freundlich isotherm model. The *R*^2^ values were found to be 0.935, 0.934 and 0.951 for AV1, AG16 and AR18, respectively, while $R_{adj}^{2}$ was equal to 0.925 for AV1 and AG16 and 0.944 for AR18. Parameters 1/*n* were in the range of 0.33–0.59 and signify that the sorption of acid dyes is favorable because the value of 1/*n* < 1. The above parameters also designate that the adsorption is multilayer and heterogeneity of adsorption sites is observed. The highest value of $k_{F}$ was calculated for AR18 dye (36.3 mg^1−1/n^ L^1/n^/g). Comparing obtained results with published ones, Kyzioł-Komosińska et al. [21] confirmed that AG16 adsorption onto natural and modified clay obeys the Freundlich model (*R*^2^ = 0.9299–0.9892, 1/*n* < 1, $k_{F}$ = 0.0811–0.1475 mg^1−1/n^ L^1/n^/g). The same conclusions were reported by Chaleshtori et al. [22], investigating AR18 adsorption on activated charcoal prepared from almond shell. Lu et al. [23] described AR18 retention by Fe_3_O_4_/MRCS (composite of melamine-formaldehyde resin with chitosan) at various temperatures and found it favorable (*R*^2^ values calculated from Freundlich isotherms were in the range 0.8017–0.9677); however, the Redlich-Peterson isotherm fit the adsorption process best.

In the case of the Langmuir model, the coefficients of determination (and adjusted, *R*^2^) were observed to be very low: 0.661, 0.665 and 0.895 for AV1, AG16 and AR18, respectively. The monolayer sorption capacities (*Q*_0_) equal 58.3 mg/g for AV1, 129.2 mg/g for AG16 and 143.7 mg/g for AR18 and do not coincide with the maximum sorption capacities values obtained under experimental conditions (*Q_e exp_*). 

The maximum sorption capacities calculated from the Dubinin-Radushkevich model (*q_m_* = 33.67–64.7 mg/g) do not match the experimental data either. The estimated values of the mean free energy *E* indicated that the sorption of the acid dyes on DVB-*co*-GMA-TETA is of a physical nature (*E* < 8 kJ/mol) [21]. However, the Dubinin-Radushkevich model can not be applicable in the description of investigated systems as equilibrium data did not show a good fit to the model (*R*^2^ = 0.340–0.626, $R_{adj}^{2}=0.246-0.573$). 

Relatively low values of the determination coefficients are observed in the dyes–DVB-*co*-GMA-TETA systems using non-linear fitting of the experimental data to the Temkin model (*R*^2^ = 0.884–0.934). Table 4 allows the comparison of the obtained data with published ones concerning removal of acidic dyes on different adsorbents.

**Figure 6:**
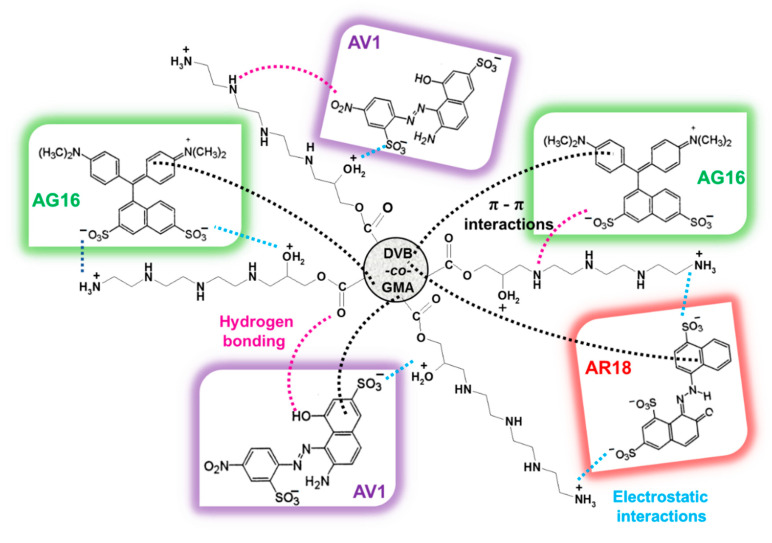
Suggested mechanism of dye interactions with DVB-*co*-GMA-TETA in aqueous solution.

The dye adsorption on DVB-*co*-GMA-TETA probably takes place as a result of physisorption and chemical interactions that were confirmed by the ATR-FT-IR analysis. The electrostatic interaction (ionic bond) between the positively charged functional groups of the resin and the negatively charged sulfonic groups of the dyes are created [24]. Additionally, hydrogen bonds and π-π interactions between the aromatic rings of benzene present in the resin matrix and dyes may occur. Figure 6 presents a probable mechanism of the dyes bonding by the resin. 

### 2.3. Determination of Kinetic Parameters

The mechanism of dye adsorption generally can involve three steps, one or any combination of which can be the rate-controlling ones: (i) mass transfer through the external boundary layer of liquid surrounding the microspheres; (ii) adsorption on the surface (internal or external), the energy depending on the type of binding process (physical or chemical)—this step is often extremely rapid; (iii) diffusion of the dye molecules to the adsorption sites either by a pore diffusion process or by a solid surface diffusion mechanism. The contact time-dependent experiments in the dyes–DVB-*co*-GMA-TETA systems were conducted to evaluate the kinetic parameters of sorption. The amounts of acid dye uptake by microspheres were calculated from Equation (6) as follows:

(6) $$q_{t}=\;\frac{\left(C_{0}-\;C_{t}\right)}{m}\;V$$

where *C*_0_ and *C_t_* (mg/L) are acid dye concentrations in the solution before adsorption and after sorption time *t*, respectively; *V* (L) is volume of the dye solution and *m* (g) is mass of DVB-*co*-GMA-TETA.

The kinetics of AV1, AG16 and AR18 adsorption on DVB-*co*-GMA-TETA were examined to explore the dye retention rate-controlling mechanism using the pseudo-first order (PFO), the pseudo-second order (PSO) and intraparticle diffusion (IPD) kinetic models [30,31]. The equations of the above mentioned models (Equations (7)–(9)) are expressed as:

(7) $$\frac{dq_{t}}{dt}=k_{1}\left(q_{e}-q_{t}\right)$$

(8) $$\frac{dq_{t}}{dt}=\;k_{2}{\left(q_{e}-q_{t}\right)}^{2}$$

(9) $$q_{t}=\;k_{i}t^{1/2}$$

where *q_e_* and *q_t_* (mg/g) are dye amounts sorbed at the equilibrium and at any time *t, k*_1_** (1/min) and *k*_2_ (g/mg min) are rate constants of sorption determined from PFO and PSO equations, respectively, and *k_i_* is the intraparticle diffusion rate constant (mg/g min^0.5^).

**Figure 7:**
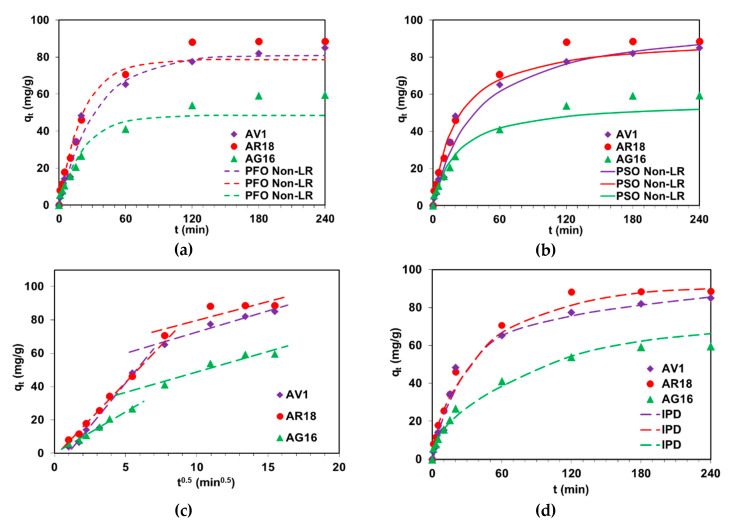
Influence of phase contact time on the acid dye uptake (*C*_0_ = 100 mg/L) by DVB-*co*-GMA-TETA with non-linear (Non-LR) fitting of PFO (**a**) and PSO (**b**) kinetic models to experimental data as well as IPD multilinear plot (**c**) and the linear fitting of IPD (**d**) model to experimental data.

There are two essential steps in the adsorption of dyes onto DVB-*co*-GMA-TETA. The first one covers the time from 1 min to 120 min and is related to the visible increase in the amount of dyes adsorbed per unit mass of the resin. The second one covers the subsequent minutes (from 120 to 180 min), in which the adsorption progress is much slower and leads to the establishment of a dynamic equilibrium (Figure 7). After 180 min, the AV1, AG16 and AR18 adsorption reached equilibrium and the maximum dye adsorption was 85 mg/g, 59.9 mg/g and 88.7 mg/g, respectively. The non-linear plots (Figure 7a,b) showing the fit of the PFO and PSO models for the sorption of AV1, AG16 and AR18 on DVB-*co*-GMA-TETA were obtained and the calculated kinetic parameters are presented in Table 5.

The determination coefficients *R*^2^ (and $R_{adj}^{2}$) of the PFO and PSO models were in the range of 0.916–0.991 (0.892–0.987) and 0.959–0.989 (0.947–0.986), respectively. Moreover, *q_e_* calculated from the PFO and PSO equations are not in line with experimental values (*q_exp_*). It can be concluded that neither PFO nor PSO models can be applied for description of kinetic sorption data of the acid dyes on DVB-*co*-GMA-TETA. However, these models are frequently used for calculation of kinetic parameters during dye removal technologies (Table 4). It was observed that the intraparticle diffusion may be a rate-limiting step, as the *R*^2^ and $R_{adj}^{2}\;$ values were high and equaled 0.939 and 0.922, 0.991 and 0.990 as well as 0.989 and 0.986 for AV1, AR18 and AG16 sorption on DVB-*co*-GMA-TETA, respectively. The best fitting of experimental data to the IPD model was obtained as shown in Figure 7d. The intraparticle diffusion rate constants calculated from the first part of multilinear plot *q_t_* vs *t*^0.5^ (Figure 7c) were 10.39 mg/g min^0.5^ for AV1, 8.88 mg/g min^0.5^ for AR18 and 4.59 mg/g min^0.5^ for AG16. 

### 2.4. Auxiliaries Presence on Dye Adsorption Effectiveness

**Figure 8:**
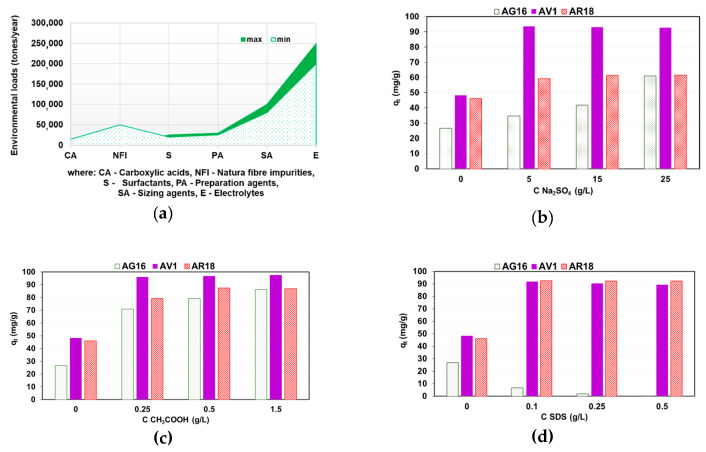
Environmental loads of auxiliaries in European countries (**a**) [10] and impact of Na_2_SO_4_ (**b**), CH_3_COOH (**c**) and anionic surfactant SDS (**d**) on AV1, AG16 and AR18 (C_0_ = 100 mg/L) sorption on DVB-*co*-GMA-TETA.

The assessment of the influence of additives (called auxiliaries in the chemical processing of textiles) present in dyeing baths and textile effluents on the effectiveness of dye retention is an extremely important part of the experiments. Auxiliaries are used in large amounts to facilitate the binding of dyes to the fabric and they get into the sewage in the amounts used for dyeing, significantly modifying the composition of the sewage. On the basis of [10], the amounts of the additives such as acids, salts and surfactants detected in the sewage of the textile industry in Europe are presented in Figure 8a. Therefore, it was decided to study their effect on the sorption capacity of DVB-*co*-GMA-TETA microspheres. In the present study, the impact of auxiliaries such as Na_2_SO_4_ (5–25 g/L), CH_3_COOH (0.25–1.5 g/L) and anionic surfactant sodium dodecyl sulfate (SDS; 0.1–0.5 g/L) were investigated on the dye (*C*_0_ = 100 mg/L) uptakes (Figure 8b–d).

Increasing the amount of Na_2_SO_4_ and CH_3_COOH in the systems caused an increase of the *q_t_* values. The amount of AG16 sorbed by the DVB-*co*-GMA-TETA increased from 26.7 mg/g to 61.1 mg/g with increasing concentration of Na_2_SO_4_. An almost two-fold increase in the amount of AV1 adsorbed by the resin (*q_t_* = 93.5 mg/g) in the 5–25 g/L Na_2_SO_4_-100 mg/L AV1 system was noted compared to solutions without electrolyte (*q_t_* = 48.3 mg/g). This can be explained by the salting out effect. The solubility of the dyes in the water phase changes, which favors its adsorption in the resin phase. It was found that in the presence of CH_3_COOH, the amount of dyes retained by the microspheres increased significantly (from 26.7 to 86.4 mg/g for AG16, from 48.3 to 97.5 mg/g for AV1 and from 46.1 to 86.9 mg/g for AR18). In acidic medium, protonation of the amine functionalities occurs and the interaction between dye anions and positively charged groups of the resin is enhanced. Such observations were described in the case of the azo dye adsorption on polystyrene and polyacrylic anion exchangers [32,33], C.I. Acid Orange 10 on an anion exchange resin (crosslinked acrylic copolymer based on divinylbenzene, ethyl acrylate and acrylonitrile functionalized with triethylenetetramine) [34].

Analyzing the adsorption efficiency of the azo dyes, i.e., AV1 and AR18, their enhanced retention on the resin in the presence of the anionic surfactant SDS was observed, while the adsorption of the triarylmethane dye AG16 decreased significantly with increasing SDS content in the system from 0.1 to 0.5 g/L. In the case of AV1 and AR18 adsorption, hydrophobic interactions between aromatic rings in dye anions and matrix of the resin play a dominant role in the retention mechanism. Electrostatic interaction between AG16 bearing positive charge and anionic SDS occurred, resulting in a reduction of its concentration in the solution and the formation of dye complexes with the surfactant, which in turn reduces its adsorption in the resin phase [33,35,36,37]. It is important to know the interactions between dyes and surfactants, especially anionic ones, which can compete with dyes of active sites of adsorbents and influence adsorption capacity of resins. 

### 2.5. Regeneration Studies

**Figure 9:**
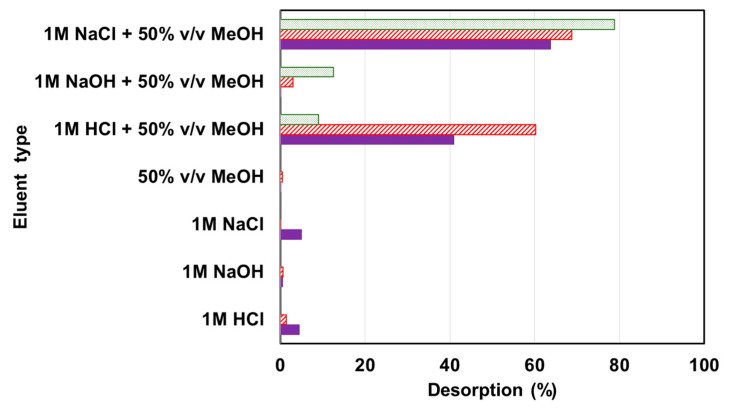
Regeneration of DVB-*co*-GMA-TETA.

In order to make the adsorption process ecologically and economically feasible, the regeneration studies should be performed to establish adsorption capability of the resin. Methanol addition greatly improves desorption efficiency of dyes (Figure 9), which proves a mixed mechanism of dye retention. For AV1, AR18 and AG16, 63.8%, 68.9% and 78.9% were desorbed, respectively, using 1 M NaCl in 50% methanol. The solution 1 M HCl in 50% methanol used for resin regeneration provided lower desorption efficiency (from 9.1% to 60.3%). The same observation was made by Xing et al. [28] investigating desorption of AR18 from the anion exchange membrane; 90% of AR18 was desorbed using 1 M NaCl in 60% *v/v* ethanol.

## 3. Materials and Methods

### 3.1. Chemicals and Eluents

**Figure 10:**
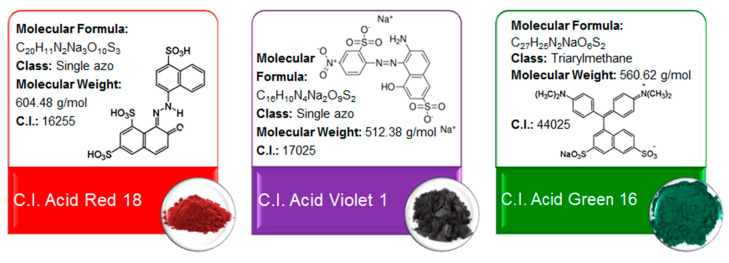
Dye characteristics.

Three textile acid dyes such as C.I. Acid Red 18, C.I. Acid Violet 1 and C.I. Acid Green 16 were purchased from Boruta-Zachem (Zgierz, Poland). Their physicochemical properties are presented in Figure 10. Acid dyes, named for their application under acid conditions, are reasonably easy to apply, have a wide range of colors and, depending on dye selection, can have good color fastness properties. They are widely applied for wool, silk and nylon dyeing.

Glycidyl methacrylate (GMA), polyvinyl alcohol (APV, MW = 72,000, 98% degree of hydrolysis), decan-1-ol and triethylenetetramine (TETA) were purchased from Fluka AG (Munich, Germany).

α,α’-azoisobisbutyronitrile and divinylbenzene (62.2% of 1,4-divinylbenzene, 0.2% of 1,2-divinylbenzene and ethylvinylbenzene) were obtained from Sigma-Aldrich (Darmstadt, Germany). Acetone, toluene, methanol, acetic acid, sodium dodecyl sulfate, sodium sulfate, sodium chloride, sodium hydroxide and hydrochloric acid were obtained from Avantor Performance Materials Poland S.A. (Lublin, Poland). Purified water came from Millipore (UMCS, Lublin, Poland). 

### 3.2. Preparation of Microspheres 

The synthesis of microspheres was performed in the aqueous medium using suspension polymerization methodology [13,14]. Redistilled water (150 mL) and 1.5 g of APV were stirred for 1 h at 80 °C in a three-necked flask fitted with a water condenser, a stirrer and a thermometer. Then the solution containing 10 g of DVB and 10.92 g of GMA (1:1 molar mass), 1% (*w/w*) of initiator (α,α’-azoisobisbutyronitrile) and the mixture of pore-forming diluents (toluene 10 mL and decan-1-ol 10 mL) were added (while stirring) to the aqueous medium. Copolymerization was completed for 10 h at 80–85 °C. The obtained microspheres were washed with distilled water (2 L), filtered off, dried and extracted in a Soxhlet apparatus with boiling acetone (5 h). Microscopic examination showed that, in most cases, particles of spherical shapes with diameters in the range 80–120 µm were obtained (*L_ep_* = 1.7 mmol/g).

### 3.3. Functionalization

The DVB-*co*-GMA copolymer was modified with TETA in the epoxide ring-opening reaction [10,11]. In a 250 mL round-bottom two-necked flask equipped with a mechanical stirrer and a thermometer, 10 g of selected microspheres were placed together with triethylenetetramine (TETA) (ten-fold excess to epoxide groups) and 200 mL of toluene. The whole content was heated over at 80 °C for 6 h and then for 8 h at room temperature. The modified microspheres were washed with distilled water (1 L), filtered off, dried and extracted in a Soxhlet apparatus with boiling toluene for 3 h. Next, the product was dried at 50 °C in a cheated chamber. The yield of reaction was 95%.

### 3.4. Characterization

The CHN elemental analysis was made using a Vario EL III analyzer (Elementar Analysensysteme GmbH, Germany). The spherical shape and morphologies of the polymeric microspheres were studied using optical microscope MORPHOLOGI G3 (Malvern, Great Britain).

Such parameters as specific surface areas, pore volumes, average pore diameters and pore size distributions were determined by the method of nitrogen adsorption on the surface of the parent DVB-*co*-GMA microspheres in a dry state. The specific surface areas were calculated by the Brunauer–Emmett–Teller (BET) method, assuming that the area of a single nitrogen molecule is 16.2 Å^2^. The pore volumes and pore size distributions were determined by the Barrett-Joyner-Halenda (BJH) method. These determinations were prepared using an adsorption analyzer ASAP 2405 (Micrometrics Inc., Norcross, GA, USA).

The attenuated total reflection (ATR) spectra were recorded using infrared Fourier transform (FT-IR) spectroscopy on a TENSOR 27 (Bruker GmbH, Leipzig, Germany) equipped with a diamond crystal. The spectra were recorded in the range of 600–4000 cm^−1^, resolution of 4 cm^−1^, 32 scans.

### 3.5. Adsorption Experiments

The stock solutions of AV1, AG16, AR18 (*C*_0_ = 1000 mg/L) were prepared and the required concentrations were obtained by diluting using volumetric flasks. In the batch studies, influences of dye concentration, phase contact time, auxiliaries presence (salt: Na_2_SO_4_, acid: CH_3_COOH, surfactant: SDS) were investigated as factors governing the dye adsorption process. Concentrations of dyes and auxiliary substances in aqueous solutions were selected in such a way as to reflect their content in dyeing baths and sewage [38,39]. All adsorption experiments were performed at 25 °C using laboratory shaker Elpin 358+ (Lubawa, Poland) at 180 rpm, amplitude 8 and at the natural pH of dye solutions. After separation of liquid phases by filtration, a single-beam UV-visible absorption spectrophotometer Cary 60 (Agilent, Santa Clara, CA, USA) was used for determination of dye concentrations at the maximum absorbance wavelengths. All adsorption experiments were performed in triplicate with reproducibility ± 3%. The mean values of the results have been used for data evaluation. A non-linear method for calculation of isotherm and kinetic parameters in adsorption studies was applied using Microsoft Excel 2013 Solver software add-in. Analysis of the Marquardt’s percent standard deviation (MPSD), determination coefficient (*R*^2^) and adjusted R-squared ($R_{adj}^{2}$) values was suggested to find the best fitting model. Adjusted R-squared is usually used to compare models of different sizes; in contrast to *R*^2^, it can go up or down when a variable is taken out of the model. The above mentioned parameters can be determined using Equations (10)–(12) [40,41]:

(10) $$MPSD=\;\overset{n}{\underset{i=1}{\sum }}{\left(\frac{q_{e\;exp}-q_{e\;cal}}{q_{e\;exp}}\right)}_{i}^{2}$$

(11) $$R^{2}=1-\frac{\sum^{\;}{\left(q_{e\;exp}-q_{e\;cal}\right)}^{2}}{\sum^{\;}{\left(q_{e\;exp}-q_{e\;mean}\right)}^{2}}$$

(12) $$R_{adj}^{2}=1-\left[\frac{\left(1-R^{2}\right)\left(n-1\right)}{n-k-1}\right]$$

where *q_e exp_* (mg/g) is amount of acid dyes sorbed at equilibrium, *q_e cal_* (mg/g) is amount of acid dyes sorbed calculated from the non-linear models, *q_e mean_* (mg/g) is measured by the mean of *q_e exp_* values, *n* is number of points in data sample, *k* is number of independent regressors. 

#### 3.5.1. Determination of DVB-co-GMA-TETA Sorption Capacity 

In order to determine the sorption capacity of DVB-*co*-GMA-TETA for dyes, 0.02 g of the microspheres and 0.02 L of the previously prepared AV1, AG16 and AR18 solutions of increasing initial concentrations (from 5 to 500 mg/L) were mixed using a laboratory shaker for 24 h (equilibrium state). The adsorption capacity (*q_e_*) was calculated from mass balance using Equation (1). The Langmuir, Freundlich, Temkin and Dubinin-Radushkevich isotherm models (Table 2) were chosen for description of experimental equilibrium data.

#### 3.5.2. Kinetic Studies and Impact of Auxiliaries on Dye Uptake

Using PFO, PSO and IPD models (Equations (7)–(9)), kinetic parameters of dye sorption on the resin were calculated under the following batch experimental conditions: phase contact time *t* = 1, 3, 5, 10, 15, 30, 60, 120, 180, 240 min, dye initial concentration *C*_0_ = 100 mg/L, solution volume *V* = 0.02 L, adsorbent mass *m* = 0.02 g, amplitude and rotary of shaking: 8 and 180 rpm, respectively.

The impact of additives such as Na_2_SO_4_, CH_3_COOH and SDS on acid dye (*C*_0_ = 100 mg/L) uptake by DVB-*co*-GMA-TETA was evaluated under the above mentioned conditions in the following systems: 5 g/L, 15 g/L and 25 g/L of Na_2_SO_4_, 0.25 g/L, 0.5 g/L and 1.5 g/L of CH_3_COOH and 0.1 g/L, 0.25 g/L and 0.5 g/L of SDS. The amount of dyes sorbed by the microspheres (*q_t_*) was determined after 30 min of phase contact time.

#### 3.5.3. Resin Regeneration

The samples of resin (0.02 g) uploaded with AV1, AR18 or AG16 (*q_e_* = 50 mg/g) were shaken for 3 h with 0.02 L of eluting solutions such as 1 M NaCl, 1 M NaOH, 1 M HCl, 1 M NaCl–50% *v/v* CH_3_OH, 1 M NaOH–50% *v/v* CH_3_OH and 1 M HCl–50% *v/v* CH_3_OH. The amount of dye desorbed from the resin phase was determined in liquid phase using UV-visible measurements and calculated by mass balance as a percentage.

## 4. Conclusions

Synthesis of microspheres based on DVB and GMA functionalized with TETA was performed and the sorption properties towards azo and triarylmethane dyes such as AV1, AR18 and AG16 were evaluated using the batch method. The Freundlich isotherm model, which takes into consideration the heterogeneity of the surface of the adsorbent, can be applied for the description of equilibrium sorption data. AG16 (142.4 mg), AV1 (172 mg) and AR18 (216.3 mg) were adsorbed by DVB-*co*-GMA-TETA (1.0 g) at equilibrium at 25 °C. The dye retention mechanism involved physical and chemical interactions. The kinetic of acid dye sorption was determined by the intraparticle diffusion with *k_i_* values in the range 4.59–10.39 mg/g min^0.5^. Presence of Na_2_SO_4_ (5–25 g/L), CH_3_COOH (0.25–1.5 g/L) and SDS (0.1–0.5 g/L) has a positive effect on the adsorption of dyes, except for the AG16-SDS system, in which a drop in the amount of dye adsorbed was observed. The highest regeneration efficiency was obtained using 1 M NaCl in 50% *v/v* CH_3_OH solution. The batch test results confirmed the possibility of DVB-*co*-GMA-TETA application as an effective adsorbent for the acid dye removal from aqueous solutions and dyeing baths.

## Figures and Tables

**Table 1:** Parameters of the porous structures of the divinylbenzene copolymer with glycidyl methacrylate (DVB-*co*-GMA) and functionalized microspheres (DVB-*co*-GMA-TETA) compared with literature data.

Resin Name	Specific Surface Area S_BET_ (m^2^/g)	Total Pore Volume V_TOT_ (cm^3^/g)	Average Pore Diameter (nm)	Ref.
DVB-*co*-GMA DVB-*co*-GMA-TETA	152 139	0.524 0.596	13.82 17.00	This study
Amberlite XAD-4 (PS-DVB)	750	0.5	5.0	[15,16]
Amberlite XAD-7 (PA-DVB)	450	1.14	9.0	[16,17]
Polyaniline/SiO_2_ nano-composite Polypyrrole/SiO_2_ nano-composite	72.4 122.81	-	-	[18]
Bentonite (Mendoza, Argentina)	8.77	0.315	7.3	[19]

**Table 2:** Isotherm models applied for fitting of experimental data [20].

Equation No.	Isotherm	Non-Linear Isotherm Form	Calculated Parameters
(2)	Freundlich	$q_{e}=k_{F}C_{e}^{1/n}$	$k_{F}$(mg^1−1/n^ L^1/n^/g) and *n* are the Freundlich constants related to adsorption capability and adsorption intensity, respectively
(3)	Langmuir	$q_{e}=\frac{k_{L}Q_{0}C_{e}}{1+C_{e}k_{L}}$	*k_L_* (L/mg) is the constant parameter of adsorption equilibrium, *Q*_0_ (mg/g) is the monolayer adsorption capacity
(4)	Temkin	$q_{e}=\frac{RT}{b_{T}}lnAC_{e}$	*b_T_* (J g/mol mg) is the Temkin constant related to the heat of adsorption, *A* (L/mg) is the Temkin isotherm equilibrium binding constant
(5)	Dubinin-Radushkevich	$q_{e}=q_{m}e^{k_{DR}\varepsilon^{2}}$ $\varepsilon =RTln\left[1+\frac{1}{C_{e}}\right]$ $E=\frac{1}{\sqrt{2k_{DR}}}$	*q_m_* (mg/g) is the maximum adsorption capacity, *k_DR_* (mol^2^ J^2^) is the constant related to the adsorption energy, *ε* (J/mol) is the adsorption potential, *E* (J/mol) is the mean free energy for removing dye molecule from its adsorption site to the infinity

where *R* is the gas constant (8.314 J/mol K), *T* (K) is the temperature.

**Table 3:** Values of parameters calculated in terms of isotherm models used.

Isotherm	Parameters	Dyes		
		AV1	AG16	AR18
Freundlich	$k_{F}$(mg^1−1/n^ L^1/n^/g) 1/*n* MPSD *R*^2^ $R_{adj}^{2}$	30.5 0.33 1.487 0.935 0.925	6.7 0.59 0.359 0.934 0.925	36.3 0.36 1.472 0.951 0.944
Langmuir	*k_L_* (L/mg) *Q*_0_ (mg/g) MPSD *R*^2^ $R_{adj}^{2}$	2.8 58.3 4.165 0.661 0.612	0.030 129.2 0.536 0.665 0.617	0.77 143.7 3.482 0.895 0.880
Temkin	*b_T_* (J g/mol mg) *A* (L/mg) MPSD *R*^2^ $R_{adj}^{2}$	351.2 175.4 3.310 0.915 0.903	273 2.1 2.108 0.884 0.867	300.5 159.6 3.499 0.934 0.925
Dubinin-Radushkevich	*q_m_* (mg/g) *k_DR_* (mol^2^ J^2^) *E* (kJ/mol) MPSD *R*^2^ $R_{adj}^{2}$	64.7 3,8∙10^−8^ 3.6 7.627 0.477 0.402	33.67 1.6∙10^−6^ 0.57 3.863 0.340 0.246	63.7 3.6∙10^−8^ 4.4 6.350 0.626 0.573

**Table 4:** The kinetic and equilibrium parameters of AV1, AG16 and AR18 sorption on various adsorbents based on the literature review.

Sorbent	Kinetic Studies	Equilibrium Studies	Ref.
AV1			
3-aminopropyl-triethoxysilane N-2-(aminoethyl)-3-aminopropyltrimethoxysilane	Disposal extent: 52–99.6% Disposal extent: 79–99.8%	-	[24]
**AG16**			
Magnetic geopolimer	PSO, *k*_2_ = 0.001–0.005 g/mg min, pH = 2.3, a.d = 0.75 g/L	*q_e_* = 108 mg/g, T = 25 °C, pH = 2.3, a.d. = 0.75 g/L	[25]
Low-moor peat and smectite clay	PSO, *k*_2_ = 0.0086–0.010 g/mg min, a.d. = 1 g/20 mL	*q_e_* = 12.07–13.0 mg/g, a.d. = 1 g/20 mL T = 25 °C, pH = 6.23–6.59	[13]
Molecularly imprinted polymers (MIP)	86% of AG16 was bound on the MIP in 60 min	*q_e_* = 6.9 mg/g	[26]
RBAC (rice bran-based activated carbon)	AG16 removal: 85.52–99.22% pH = 3.5, T = 20–55 °C, a.d. = 1 g/50 mL	*q_e_* = 1.05–1.36 mg/g, T = 20–55 °C, pH = 2.0–3.5	[27]
**AR18**			
Anion exchange membrane with quaternary ammonium groups (SB 6407)	PSO, *h* = 4.3 mg/g min^1/2^	*q_e_* = 217.6 mg/g, T = 25 °C, pH = 7	[28]
Wool powder (p.s. = 6.2 μm, s.a. = 5.91 m^2^/g)	AR18 removal: 90%, pH = 2.5	*q_e_* = 100 mg/g, T = 25 °C, pH = 7	[29]

where *q_e_* is equilibrium capacity, a.d. is adsorbent dosage, *k*_2_ is pseudo-second order rate constant, *h* is initial adsorption rate constant, p.s. is particle size, s.a. is surface area.

**Table 5:** Kinetic parameters of sorption determined in the 100 mg/L AV1/AG16/AR18–DVB-*co*-GMA-TETA systems.

Parameter	Dye		
	AV1	AR18	AG16
*q_exp_* (mg/g)	85.0	88.7	59.9
**PFO**			
*q_e_* (mg/g)	80.9	78.6	48.5
*k*_1_ (1/min)	0.0299	0.0464	0.0450
MPSD	0.361	0.499	0.689
*R* ^2^	0.991	0.951	0.916
$R_{adj}^{2}$	0.987	0.936	0.892
**PSO**			
*q_e_* (mg/g)	100.6	91.3	56.5
*k*_2_ (g/mg min)	0.0003	0.0005	0.0008
MPSD	0.339	0.326	0.462
*R* ^2^	0.989	0.977	0.959
$R_{adj}^{2}$	0.986	0.971	0.947
**IPD**			
*q_e_* (mg/g)	85.6	66.3	90.01
*k_i_* (mg/g min^0.5^)	10.39	8.88	4.95
*R* ^2^	0.939	0.991	0.989
$R_{adj}^{2}$	0.922	0.990	0.986
